# Child and Adolescent Psychiatry and Mental Health reviewer acknowledgement 2013

**DOI:** 10.1186/1753-2000-8-4

**Published:** 2014-02-05

**Authors:** Jörg M Fegert

**Affiliations:** 1 Department of Child and Adolescent Psychiatry and Psychotherapy, University of Ulm, Steinhoevelstr. 5, Ulm 89075, Germany

## Abstract

**Contributing reviewers:**

The editors of *Child and Adolescent Psychiatry and Mental Health* would like to thank all of our reviewers who have contributed to the journal in volume 7 (2013).

## 

Gwen Adshead

United Kingdom

Alka Ahuja

United Kingdom

Kenneth Aitken

United Kingdom

Celso Arango

Spain

Filip K. Arnberg

Sweden

Philippe Auby

France

Agnes Ayton

United Kingdom

Josephine Barbaro

Australia

Claus Barkmann

Germany

Christopher Barry

United States of America

Jessica Beatty

United States of America

Katja Becker

Germany

Morton Beiser

Canada

Myron Belfer

United States of America

Frits Boer

Netherlands

Maria Teresa Botello-Harbaum

United States of America

Ina Bovenschen

Germany

Heloisa Brasil

Brazil

Patricia Brennan

United States of America

Per Hakan Brondbo

Norway

Anat Brunstein Klomek

United States of America

Brendan Bunting

United Kingdom

Larry Burd

United States of America

Marcy Burstein

United States of America

Luna Centifanti

United Kingdom

David Chambers

United States of America

Alexander Chapman

Canada

Deena Chisolm

United States of America

Molly Choate Summer

United States of America

Lucia Ciciolla

United States of America

Paula Cloutier

Canada

Judith Cohen

United States of America

Delphine Collin-Vezina

Canada

Mark Connolly

Netherlands

Trudi Cooper

Australia

Robert Courtois

France

Holger Cramer

Germany

Zuzana Dankulincova Veselska

Slovakia

Deborah Davis

United States of America

William Daviss

United States of America

Domenico De Berardis

Italy

Mieke Decuyper

Belgium

Denis Köhler

Germany

John Diamond

United States of America

Arden Dingle

United States of America

Ralf W. Dittmann

Germany

Daphna Dollberg

Israel

Daniel Fatori

Brazil

Armando Favazza

United States of America

Dineke Feenstra

Netherlands

Minne Fekkes

Netherlands

Maite Ferrin

United Kingdom

David Finkelhor

United States of America

Carol Fitzpatrick

Ireland

Bacy Fleitlich-Bilyk

Brazil

Paul Frewen

Canada

Daniel Fung

Singapore

Abbe Garcia

United States of America

Kathoiki Georgiades

Canada

Grant Gillett

New Zealand

Grazina Gintiliene

Lithuania

Daniel Gorman

Canada

Taveeshi Gupta

United States of America

Lisa Harryson

Sweden

Nina Heinrichs

Germany

Marisa Hilliard

United States of America

Christopher Hopwood

United States of America

Stephanie Hullmann

United States of America

Tina In-Albon

Germany

Vanessa Jantzer

Germany

Sandra Jee

United States of America

Tine K. Jensen

Norway

Michael Kaess

Germany

Niranjan Karnik

United States of America

Ferdinand Keller

Germany

Sarah Kelly

United States of America

Stephan Kupferschmid

Switzerland

Joshua Langberg

United States of America

Kate Langley

United Kingdom

Nora Lee

United States of America

Ann Lendrum

United Kingdom

Adam Lewin

United States of America

Stephen P. Lewis

Canada

Garth Lipps

Jamaica

Arnold Lohaus

Germany

Audhild Løhre

Norway

Kerstin Malmberg

Sweden

Ivo Marx

Germany

Jon McClellan

United States of America

John McMillan

New Zealand

Lauren Miller-Lewis

Australia

Suneeta Monga

Canada

Megan Moreno

United States of America

Tally Moses

United States of America

Brenda Myles

United States of America

David Ndetei

Kenya

Wendy Nilsen

United States of America

Jessica Noggle

United States of America

Lene Nyboe

Denmark

Carsten Obel

Denmark

Ingrid Landgraff Oestlie

Norway

Atte Oksanen

Finland

Thomas Olino

United States of America

Yoon Phaik Ooi

Singapore

Sylvia Oswald

Germany

Sarah Parsons

United Kingdom

Juan Pedraza

United States of America

Eva Penelo

Spain

Noemí Pereda

Spain

Dominic Plant

United Kingdom

Paul L. Plener

Germany

Maria Cristina Porfirio

Italy

Maj-Britt Posserud

Norway

Christian Postert

Germany

Erica Prentkowski

United States of America

Colin Pritchard

United Kingdom

Nicola Reavley

Australia

Matthias Reitzle

Germany

Erin Reuther

United States of America

Joseph Rey

Australia

Michael Robertson

Australia

Thiago Rocha

Brazil

Rachel Rodgers

United States of America

Paul Rohde

United States of America

Michael Sachse

Germany

John Sadler

United States of America

Björn Salomonsson

Sweden

Caferi Tayyar Sasmaz

Turkey

Ingo Schäfer

Germany

Leonhard Schilbach

Germany

Silvia Schneider

Germany

Ulrike Margarete Elisabeth Schulze

Germany

Dorothy Seals

United States of America

Margaret Sibley

United States of America

Inga Dora Sigfusdottir

Iceland

Johan Simons

Belgium

Petros Skapinakis

Greece

John Smythies

United States of America

Gottfried Spangler

Germany

Anthony Spirito

United States of America

Marlys Staudt

United States of America

Hans-Christoph Steinhausen

Denmark

Stefan Stieger

Austria

Lisanne Stone

Netherlands

Stephen Sulzbacher

United States of America

Xiang Sun

United Kingdom

Toshinobu Takeda

Japan

Jessica Tearne

Australia

John Teshima

Canada

Ute Thyen

Germany

Maggie Toplak

Canada

Cristiano Tschiedel Belem da Silva

Brazil

Adriana Umana-Taylor

United States of America

Julee Waldrop

United States of America

Jason Washburn

United States of America

Alan Waterman

United States of America

Michelle Wedig

United States of America

Yifeng Wei

Canada

Miranda Wolpert

United Kingdom

Bernardine Woo

Singapore

Sandy Wurtele

United States of America

Yi Zheng

China

Tanja Zimmermann

Germany

Julie Zito

United States of America

